# Fine needle aspiration cytology of primary thyroid lymphoma: a report of ten cases

**DOI:** 10.1186/1742-6413-2-21

**Published:** 2005-12-09

**Authors:** Nalini Gupta, Raje Nijhawan, Radhika Srinivasan, Arvind Rajwanshi, Pinaki Dutta, Anil Bhansaliy, SC Sharma

**Affiliations:** 1 Department of Cytopathology, Postgraduate Institute of Medical Education and Research, Chandigarh, 160012, India; 2 Department of Endocrinology, Postgraduate Institute of Medical Education and Research, Chandigarh, 160012, India; 3 Department of Radiotherapy, Postgraduate Institute of Medical Education and Research, Chandigarh, 160012, India

**Keywords:** Primary thyroid lymphoma, Fine needle aspiration cytology, Prognosis.

## Abstract

Primary lymphoma is an uncommon malignancy of the thyroid, comprising of 0.6 to 5 per cent of thyroid cancers in most series. Primary thyroid lymphomas (PTL) occur most commonly in elderly women and are commonly of B- cell origin. These frequently present in clinical stage IE and IIE. We report here ten cases of PTL diagnosed over a period of about 7 years in our institute. Out of these ten cases, nine were diagnosed on fine needle aspiration cytology (FNAC) and one case was misdiagnosed as lymphocytic thyroiditis. This case was diagnosed as Non- Hodgkin's lymphoma on surgical specimen. Five patients are disease free and doing well, while two died of disease and the other two were lost to follow-up. One patient is currently on chemotherapy. The salient clinical, biochemical, radiological features, FNA findings along with diagnostic difficulties are discussed.

## Introduction

Primary thyroid lymphoma is a rare disease that continues to produce diagnostic and therapeutic dilemmas. Most thyroid lymphomas are of B-cell origin [1]. There appear to be two distinct clinical and prognostic groups of these rare tumors. The more common subtype, comprising of up to 70% of cases, is a diffuse large B-cell lymphoma [1]. This subtype appears to have the most aggressive clinical course with almost 60% of these tumors diagnosed with disseminated disease. The other subtype is mucosa-associated lymphoid tissue (MALT) lymphomas comprising of approximately 6% to 27% of thyroid lymphomas [1]. These have a relatively indolent course. These occur more commonly in females with female to male ratio of 2–4:1. The majority of these patients have underlying Hashimoto's thyroiditis, which increases the risk for thyroid lymphoma by 50 times [2]. The importance of recognizing primary thyroid lymphoma lies in the fact that this disease is quite curable without the need for extensive surgery if recognized early and treated appropriately. In this report, we discuss FNA findings of ten such patients.

## Materials and methods

Ten cases of primary thyroid non-Hodgkin's lymphoma (PTL) were retrieved from files of department of Cytopathology, Postgraduate Institute of Medical Education and Research, Chandigarh, India over a seven year period (Jan 1998- Oct 2004), during this period about 4500 patients were aspirated for thyroid enlargement. Clinical information including age, sex, presenting symptoms, treatment and subsequent course were recorded. FNAC was performed with 23 G needle and on an average 2–3 passes were taken to obtain adequate material for diagnosis. Histopathology of the surgically resected specimens was available in three cases. Immunocytochemistry (ICC) was performed for leukocyte common antigen (LCA), cytokeratin, CD 20 and CD3 in six cases.

## Results

Of the 10 cases, 5 were female and 5 were males with female to male ratio of 1: 1. The age ranged from 10–61 years with a mean age of 39.6 years. Two patients were less than 15 years of age. Duration of follow-up ranged from 1 month to 4.3 years. A summary of clinical information and cytological findings is shown in Table 1. All patients presented with thyroid enlargement of variable duration (20 days to 2 years). Seven patients presented with a diffuse goiter, two patients had a multinodular goiter and only one patient presented as a solitary thyroid nodule. Other signs and symptoms included stridor, hoarseness, dyspnoea, dysphagia and sense of heaviness in the neck. Four patients had associated cervical lymphadenopathy and three of them had lymphomatous involvement while one had reactive hyperplasia.

**Table 1:** showing clinical presentation, biochemical investigations and follow- up of ten cases of primary thyroid lymphoma.

**No**	**Age/ Sex**	**Clinical Presentation**	**Biochemistry Anti thyroid antibody**	**Diagnosis**	**Follow- up**	**Prognosis**
**1**	61 F	Nodular goiter- 2 months	Hypothyroid TMA +	NHL – HG	3 yrs	Disease free
**2**	48 F	Diffuse goiter- 20 months	Hypothyroid TMA +	FNA- LT Surgery- NHL	Lost to follow- up	
**3**	44 M	Diffuse goiter- 24 months	Euthyroid TMA +	NHL – IG	4.3 years	Disease free
**4**	60 F	Diffuse goiter- 1 month, local lymph nodes	Euthyroid TMA +	NHL – IG	4 years	Local nodal recurrence after 1 1/2 years- RT given, Stable.
**5**	13 M	Diffuse goiter- 6 months	Euthyroid TMA +	NHL – HG	3 years	Disease free
**6**	10 M	Diffuse goiter, stridor- 2 months, local lymph nodes	Euthyroid TMA -ve	NHL – HG	6 months	Lost to follow- up, Came after 6 months with SVC syndrome, **Died**.
**7**	60 M	Solitary nodule- 12 months	Hypothyroid TMA +	NHL – IG	6 months	Disease free
**8**	20 M	Diffuse goiter, Hoarse voice, stridor- 6 months, local lymph nodes	Euthyroid TMA -ve	NHL – HG	1 year	**Died **of disseminated disease
**9**	32 F	Nodular goiter, local lymph nodes- 5 months	Euthyroid TMA -ve	NHL – HG	1 month	On treatment- chemotherapy
**10**	48 F	Diffuse goiter, stridor- 20 days	Euthyroid TMA -ve	NHL – HG	Lost to follow- up	

**Figure 1:**
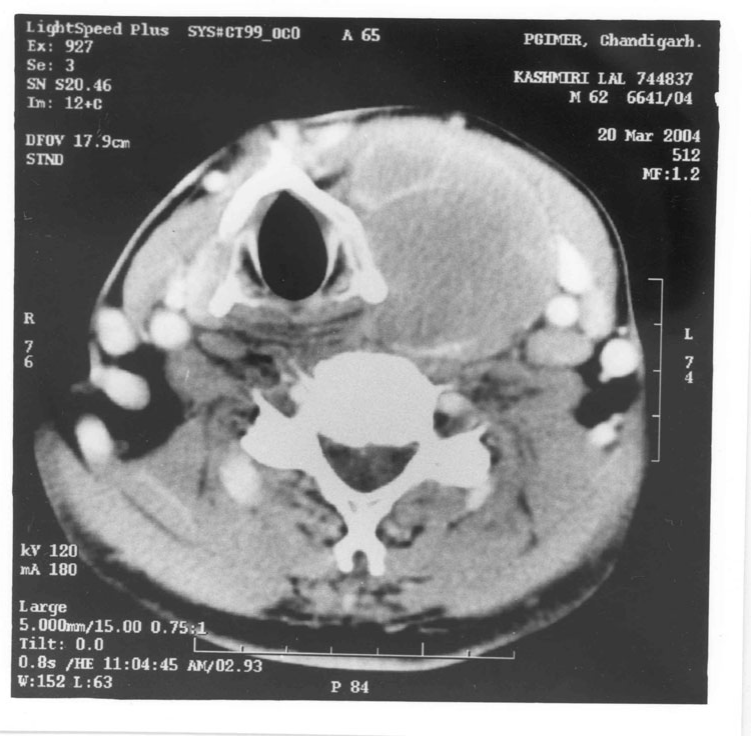
CECT scan showing a homogenous isodense soft tissue mass (3.2 × 4.5 cm) in the region of left lobe of thyroid.

Imaging modalities and bone marrow examination did not reveal other areas of involvement. CT scan done in these cases revealed a lesion primarily involving the thyroid gland (Figure 1). Hematological parameters, serum biochemistry and thyroid function tests were normal at presentation except in three patients, who were hypothyroid. Thyroid microsomal antibody (TMA) titer was significantly increased (1:80) in six patients.

**Figure 2:**
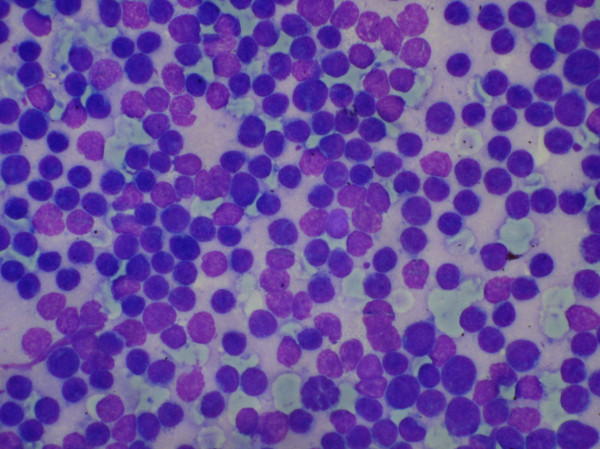
Aspiration smear showing monomorphic population of atypical lymphoid cells in a case of high grade non- Hodgkin's lymphoma (MGG × 512).

Cytologically in nine cases, a diagnosis of non- Hodgkin's lymphoma (NHL) was offered on FNAC. These included six cases of high grade NHL and three cases of intermediate grade NHL. The smears in high grade NHL cases were cellular and comprised of monomorphic population of large atypical lymphoid cells. These cells were 2–3 times the size of a mature lymphocyte with opened up chromatin and conspicuous 1–2 nucleoli (Figure 2). In the background many lymphoglandular bodies were seen. One of these six cases had subsequent histological confirmation.

**Figure 3:**
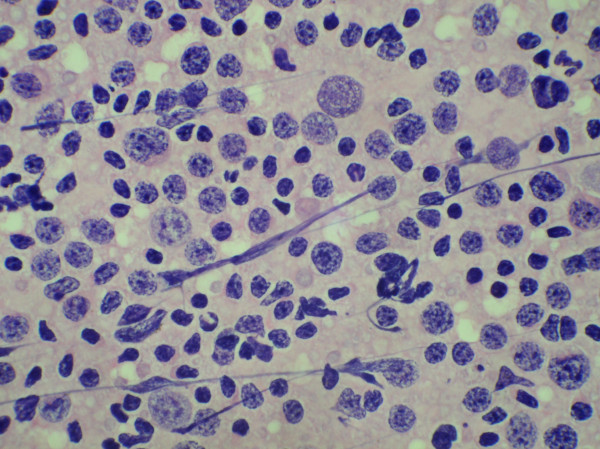
Aspiration smear showing a typical dual population of lymphoid cells in a case of intermediate grade non- Hodgkin's lymphoma (H&E × 512).

In three cases, FNAC smears showed a dual population of lymphoid cells comprising of an admixture of mature lymphocytes and larger lymphoid cells. These large lymphoid cells were 2–3 times the size of a mature lymphocyte and had opened up chromatin and conspicuous nucleoli (Figure 3). Many monocytoid cells were also seen. A diagnosis of florid lymphocytic thyroiditis was considered less likely, because, in thyroiditis, a reactive population of lymphoid cells of variable sizes admixed with plasma cells and tingible body macrophages is usually present. Hence, in these three cases, combining the clinical presentation and cytological findings, a diagnosis of NHL – intermediate grade was suggested.

In one case, the diagnosis of lymphoma was missed on FNA. The patient presented with a diffuse rather than a nodular swelling and cytology revealed a polymorphic infiltrate consistent with a reactive hyperplasia which was seen to infiltrate the follicular epithelial cell clusters. Hence a diagnosis of lymphocytic thyroiditis was offered. A subtotal thyroidectomy was performed because of associated pressure symptoms. Histopathology revealed a Non- Hodgkin's lymphoma morphologically and immunohistochemically consistent with a marginal zone B cell lymphoma.

In six cases, immunocytochemistry (ICC) was performed on the fine needle aspirate and all cases were CD 20 positive and CD3 negative.

In all the cases, protocol staging as per the ANN Arbor staging of NHL was performed. Bone marrow examination, whole body ^67^Gallium scan, CECT abdomen and chest and CSF for malignant cytology was done in all patients before labeling a case as primary thyroid lymphoma. In the present series, 7 cases were of stage I (E) and three cases were of stage II (E). Lactate dehydrogenase (LDH) levels were done in six out of ten patients and the mean LDH level was 628 U/L.

### Follow- up information

All the patients received combination chemotherapy (CHOP regime) with local radiotherapy. Five patients are alive and are free of disease till date, whereas, two patients died of the disease. These two patients had high grade lymphoma and succumbed to disseminated disease. Two were lost to follow-up and one patient is currently on chemotherapy.

## Discussion

Primary thyroid lymphoma (PTL) is defined as a lymphomatous process involving the thyroid gland without contiguous spread or distant metastases from other areas of involvement at diagnosis [3]. PTL constitutes 5% of all thyroid malignancies and occurs in less than 1% of all non- Hodgkin's lymphomas [3]. The majority of patients are middle to old aged women [4]. Studies have shown that PTL typically arises in the setting of autoimmune thyroiditis and it takes, on an average, 20 to 30 years to develop after the onset of lymphocytic thyroiditis [5]. A short history of a rapidly enlarging neck mass often associated with dyspnoea, difficulty in swallowing, or voice change is the hallmark presentation of thyroid lymphoma [6]. Therefore, clinically this may be confused with anaplastic thyroid carcinoma. Hoarseness, respiratory distress, cough and dysphagia were the usual presenting manifestations in our patients.

Hypothyroidism at the time of diagnosis is documented in 30–40% of patients due to replacement of thyroid parenchyma by the lymphomatous process or due to underlying Hashimoto's thyroiditis. Thyrotoxicosis is exceedingly rare [7]. Seven of our patients at presentation were euthyroid and three were hypothyroid. Circulating antibodies to thyroid peroxidase are positive in 60–80% of patients suggesting underlying lymphocytic thyroiditis as a predisposing factor. In the present series, six patients had significantly elevated thyroid microsomal antibody titer.

Fine needle aspiration has become the procedure of choice for the initial pathological diagnosis of thyroid nodule. However, studies have also shown inconsistent results in the diagnosis of lymphoma of the thyroid. In one series, a correct diagnosis with FNAC was made in 70–80% of patients with thyroid lymphoma [8], but in others, FNA was suggestive but not diagnostic in only 50–60% of patients [9-12]. In the present study, nine out of ten cases (90%) of the cases of PTL were correctly diagnosed by FNA. A primary thyroid non-Hodgkin lymphoma is usually of large cell type [13] and a diagnosis of large cell lymphoma is easy on FNA and features like lack of cellular cohesion and presence of lymphoglandular bodies in the background are features strongly against a diagnosis of anaplastic carcinoma [14]. ICC confirms the lymphoid origin of the cells and their B or T- lineage. By contrast, cytological diagnosis of MALT- lymphomas is difficult, because of heterogeneous appearance of the neoplastic infiltrate [14]. The principal problem with a cytological diagnosis of low- grade lymphoma of the thyroid is its differentiation from HT. The distinguishing features may be the abundance of lymphoid tissue and a high proportion of intermediate centrocyte- like cells in low- grade NHL as compared to HT. False negative results may be due to sampling error also as low- grade B- cell MALT lymphoma originates from HT and the two usually coexist [14]. Due attention to dual population of lymphoid cells, presence of monocytoid cells in FNA smears and extensive follicular epithelial destruction and the clinical setting enabled us to diagnose three cases of non- Hodgkin's lymphoma of intermediate grade. However, we missed the diagnosis of NHL on FNAC in one case reported as lymphocytic thyroiditis, which on subsequent subtotal thyroidectomy, was reported as non- Hodgkin's lymphoma. FNAC cytomorphology in conjunction with flow cytometric (FC) immunophenotyping has become a reliable and accurate method for the diagnosis and classification of many lymphoproliferative disorders. CD4/CD8 T-cell ratio comparisons are made with cytomorphological diagnoses of reactive, atypical, non-Hodgkin lymphoma, and Hodgkin lymphoma cases [15,16].

PTL frequently present in clinical stage IE and IIE. Treatment is similar to other nodal lymphoma. For patients with intermediate or high-grade lymphoma, the best results are obtained from cyclophosphamide, daunorubicin, vincrstine and prednisolone (CHOP) based chemotherapy. Radiation therapy is used most commonly after 3–6 courses of chemotherapy in form of modified mantle irradiation including thyroid, bilateral neck, supraclavicular area and mediastinum [17]. Our patients received CHOP based chemotherapy, two of our patients had relapsed with bone marrow involvement and local nodal recurrence. One died of lymphomatous process and other was controlled with radiotherapy. The poor prognostic factors include age more than 60 years, performance status grater than 1, elevated lactate dehydrogenase (LDH) and β_2 _microglobulin, extranodal sites more than 1 and Ann -Arbor staged III-IV [18,19].

In summary, we report a retrospective study of ten cases of primary thyroid lymphomas. The diagnosis was established by fine needle aspiration in nine cases and one case was misdiagnosed as lymphocytic thyroiditis, which was diagnosed on surgical specimen. The cytological diagnosis of high grade lymphoma is easy and ICC can confirm suspicious cases. The diagnosis of low grade lymphoma is more difficult but clinical and radiological suspicion and cytomorphological features can help reaching the correct diagnosis in such cases.

## Abbreviations

NHL – Non- Hodgkin's lymphoma

HG- High grade

IG- Intermediate grade

TMA- Thyroid microsomal antibody

HT- Hashimoto's thyroiditis

LT- Lymphocytic thyroiditis

RT- Radiotherapy

SVC- Superior vena cava.
